# Quantitative analysis of ChIP-seq data uncovers dynamic and sustained H3K4me3 and H3K27me3 modulation in cancer cells under hypoxia

**DOI:** 10.1186/s13072-016-0090-4

**Published:** 2016-11-01

**Authors:** Michiel E. Adriaens, Peggy Prickaerts, Michelle Chan-Seng-Yue, Twan van den Beucken, Vivian E. H. Dahlmans, Lars M. Eijssen, Timothy Beck, Bradly G. Wouters, Jan Willem Voncken, Chris T. A. Evelo

**Affiliations:** 1 Maastricht Centre for Systems Biology – MaCSBio, Maastricht University, Maastricht, The Netherlands; 2 Department of Bioinformatics – BiGCaT, Maastricht University, Maastricht, The Netherlands; 3 Department of Molecular Genetics, Maastricht University, Maastricht, The Netherlands; 4 Departments of Informatics and Bio-computing, University Health Network, Toronto, ON Canada; 5 Princess Margaret Cancer Centre and Campbell Family Institute for Cancer Research, University Health Network, Toronto, ON Canada; 6 Department of Radiation Oncology, University of Toronto, Toronto, ON Canada; 7 Maastricht Radiation Oncology (MaastRO) Laboratory, Maastricht University, Maastricht, The Netherlands; 8 Heart Centre Biobank, The Hospital for Sick Children, Toronto, ON Canada; 9 Human Longevity Inc., San Diego, CA USA

**Keywords:** Epigenomics, Transcriptomics, Data normalization, ChIP-sequencing, H3K4me3, H3K27me3, Hypoxia, MCF7

## Abstract

**Background:**

A comprehensive assessment of the epigenetic dynamics in cancer cells is the key to understanding the molecular mechanisms underlying cancer and to improving cancer diagnostics, prognostics and treatment. By combining genome-wide ChIP-seq epigenomics and microarray transcriptomics, we studied the effects of oxygen deprivation and subsequent reoxygenation on histone 3 trimethylation of lysine 4 (H3K4me3) and lysine 27 (H3K27me3) in a breast cancer cell line, serving as a model for abnormal oxygenation in solid tumors. A priori, epigenetic markings and gene expression levels not only are expected to vary greatly between hypoxic and normoxic conditions, but also display a large degree of heterogeneity across the cell population. Where traditionally ChIP-seq data are often treated as dichotomous data, the model and experiment here necessitate a quantitative, data-driven analysis of both datasets.

**Results:**

We first identified genomic regions with sustained epigenetic markings, which provided a sample-specific reference enabling quantitative ChIP-seq data analysis. Sustained H3K27me3 marking was located around centromeres and intergenic regions, while sustained H3K4me3 marking is associated with genes involved in RNA binding, translation and protein transport and localization. Dynamic marking with both H3K4me3 and H3K27me3 (hypoxia-induced bivalency) was found in CpG-rich regions at loci encoding factors that control developmental processes, congruent with observations in embryonic stem cells.

**Conclusions:**

*In silico*-identified epigenetically sustained and dynamic genomic regions were confirmed through ChIP-PCR in vitro, and obtained results are corroborated by published data and current insights regarding epigenetic regulation.

**Electronic supplementary material:**

The online version of this article (doi:10.1186/s13072-016-0090-4) contains supplementary material, which is available to authorized users.

## Background

Epigenomics explores genome-wide epigenetic modifications, such as DNA (hydroxy)methylation or posttranslational modification of N-terminal histone tails [1]; such marking is often dynamic and serves to support phenotypic plasticity. Well-known examples of histone modifications are trimethylation of histone 3 lysine 4 (H3K4me3) and lysine 27 (H3K27me3), which are often associated with transcriptional activity and repression, respectively. Combined chromatin immunoprecipitation (ChIP) and high-throughput sequencing technology (ChIP-seq [2, 3]) has proven to be a powerful approach to create genome-wide maps of such histone modifications in multiple human cancer cell lines [4–7], and the key to understanding the molecular mechanisms underlying cancer and to improving cancer diagnostics, prognostics and treatment [1, 8, 9].

In this study we focused specifically on ChIP-seq-based analysis of dynamically changing H3K4me3 and H3K27me3 enrichment in response to fluctuating oxygen levels. MCF7 cells were exposed to 8 and 24 h of hypoxia, and subsequently reoxygenated, serving as a model for such dynamic epigenetic effects occurring in response to reduced oxygen availability in solid tumors [10]. Analysis of a highly dynamic biological system, where the majority of epigenetic markings and gene expression levels will be altered between physiologically distinct states, as well as display a large degree of heterogeneity across the cell population due to epigenetic selection and stochasticity, poses a number of challenges on data analysis.

First, the expected heterogeneity, *i.e.*, the observation that the same region can harbor a specific epigenetic mark in some part of the cell population, while not in the other, results in (1) a quantitative range of ChIP-seq signals within a state and (2) quantitative differences between states. Although ChIP-seq data are traditionally treated as dichotomous (i.e., present/not present), here quantitative data comparison is therefore a must. This requires normalization of the ChIP-seq data [11]. Most commonly, a scaling approach relative to the total number of aligned reads is applied, based on the prediction that the number of differences between conditions is limited [12–14]. This assumption, however, does not hold here, as quantitative data comparison between highly variant states requires invariant measurement sets across all experimental conditions as a reference, such as an artificial spike-in [15–17], or biologically steadily marked H3K4me3/H3K27me3 regions (by analogy: steadily expressed genes in transcriptomic studies). However, a priori definition of regions with sustained epigenetic marking is challenging, as this depends on the biological model as well as the studied histone modification [13]. To accommodate study-specific approaches [18], an unsupervised approach is required to identify and define epigenetically invariant genomic regions.

Second, although biological interpretation of ChIP-seq data by itself is legitimate [19–21], integration of transcriptomics data is pivotal for a comprehensive biological interpretation [22]. We extrapolate from this observation to propose that transcriptomics data analysis is a requirement for biology-driven preprocessing of ChIP-seq data. Specifically, the invariant regions used for ChIP-seq data normalization should be defined in conjunction with transcriptomics data.

Considering the challenges presented by a highly variant biological system, we pursued the invariant-set paradigm outlined above in a data-driven analysis approach combining genes with sustained expression and genomic regions with sustained H3K4me3 and H3K27me3 markings across all states. The approach enables quantitative data comparison for H3K4me3 and H3K27me3 ChIP-seq data and a comprehensive integrative analysis of epigenomics and transcriptomics data. The biological insights derived from the analysis are further corroborated a posteriori by publicly available data and current insights regarding epigenetic regulation.

## Results

### Data generation

MCF7 cells were exposed to 8 or 24 h of hypoxia (0.02 % oxygen) and subsequently reoxygenated for 8 h (21 % oxygen) (see Methods section for details). Cells were harvested and processed for ChIP-seq and microarray-based expression analysis at 0, 8 and 24 h of hypoxia (referred to as *t* = 0, *t* = 8 and *t* = 24, respectively), and at 8 h of reoxygenation (referred to henceforth as *t* =+8).

### Quantitative analysis of sustained and dynamic H3K27me3 enrichment under fluctuating oxygen levels

To enable quantitative analysis, H3K27me3 datasets were normalized based on identification of regions with sustained H3K27me3 enrichment between all samples analyzed. The cumulative area under the curve (AUC) for all peaks in all these regions was determined for each condition, resulting in sample-specific scaling factors (see Methods section for details).

For H3K27me3, after normalization, a single cutoff value for all samples was set at the peak height above which the H3K27me3 data correlated with the ChIP-seq data of CBX8, a known H3K27me3-binding protein under normoxic conditions (*t* = 0) [23], corresponding to a normalized height of 5.0 on a log_2_ scale. For biological interpretation analyses, we set the level of biological significance at two times this background level (6.0 on a log_2_ scale) and considered peaks below this value background noise (*i.e.*, biologically irrelevant). To add further biological validity to this threshold, we defined a set of 1137 genes whose expression levels were above the 95th percentile for all samples analyzed, *i.e.*, consistently highly expressed genes. Any repressive H3K27me3 enrichment present in these genes were considered background noise, and indeed, the median enrichment of highly expressed genes H3K27me3 peaks was found to be below our previously calculated threshold (4.9 on log_2_ scale).

**Fig. 1:**
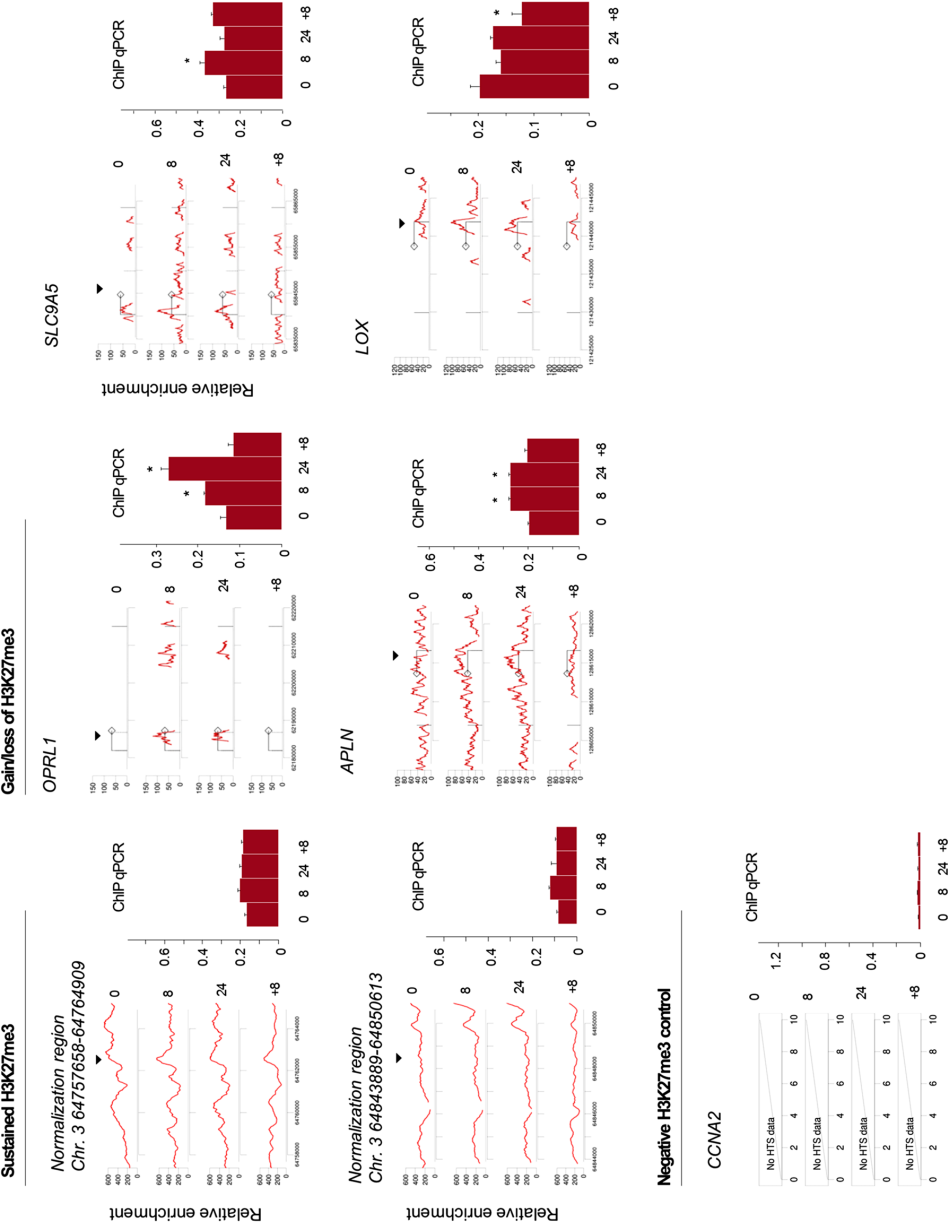
Validation of sustained and dynamic H3K27me3 marking. Bioinformatic calling of enriched regions (gene tracks shown at left side for each locus) was validated by ChIP-PCR (*bar plots*; shown at *right side* for each locus) for a number of representative epigenetic profiles. Data for all time points are shown: normoxia (0), hypoxia 8 h (8), hypoxia 24 h (24) and reoxygenation (+8). Two regions used for normalization on chromosome 3 show sustained H3K27me3 enrichment (*left panels*); the *CCNA2* locus is known to be free of H3K27me3 marking [24] (*bottom panel*); *OPRL1, SLC9A5, APLN and LOX* for genes that show dynamic modulation of H3K27me3 marking in relation to changes in oxygen pressure; gene-specific ChIP-PCR analyses are part of a biological study published elsewhere [10]. *Diamond symbol* indicates direction of transcription, *black triangle* indicates approximate position of primer set used for validation, and star symbol indicates a significant difference compared to *t* = 0 (i.e., normoxia; *p* < 0.05, Wilcoxon signed-rank test)

For the dataset used to develop the protocol, regions with sustained epigenetic marking were found near centromeres and in intergenic regions. A number of regions with dynamically altered or sustained epigenetic marking were validated by conventional ChIP-PCR (Fig. 1): Oxygen deprivation and/or reoxygenation clearly affected H3K27me3 enrichment at the *APLN* and *OPRL1* loci, whereas the two indicated regions on chromosome 3 remained relatively constant. The *SLC9A5* locus shows an increase in response to 8 h of hypoxia, followed by a drop at 24 h of hypoxia and again an increase upon reoxygenation. The ChIP-seq results suggest a slight increase in H3K27me3 enrichment around the TSS at the *LOX* locus, which is restored upon reoxygenation. In the ChIP-PCR results, only the drop in enrichment upon reoxygenation is reflected. The *CCNA2* locus was used as a negative reference as it is known to be free of H3K27me3 marking [24].

Correlation analysis revealed a substantial overlap between peaks for each trimethyl histone modification under normoxic versus hypoxic conditions (Spearman ρ at 0 versus 24 h of hypoxia: 0.77 for H3K4me3, 0.60 for H3K27me3; *p* < 0.05). These data indicated that preexistent (normoxic) H3K4me3 and H3K27me3 marking was generally retained under hypoxic conditions and that enrichment increased during hypoxia. Of relevance, these observations validated the *invariant region* normalization strategy supporting the comparative histone H3K27me3 analysis. H3K4me3 enrichment (*i.e.*, the relative amount of the genome associated with peaks) returned to the normal situation upon reoxygenation (ρ for normoxia versus reoxygenation: 0.82; *p* < 0.05). In contrast, H3K27me3 enrichment showed poor correlation between normoxia and reoxygenation (*ρ* = 0.19; not significant), indicating that the original normoxic H3K27me3 distribution had not been restored at 8 h of reoxygenation.

### Identification of genes with sustained and dynamic H3K4me3 enrichment under fluctuating oxygen levels

**Fig. 2:**
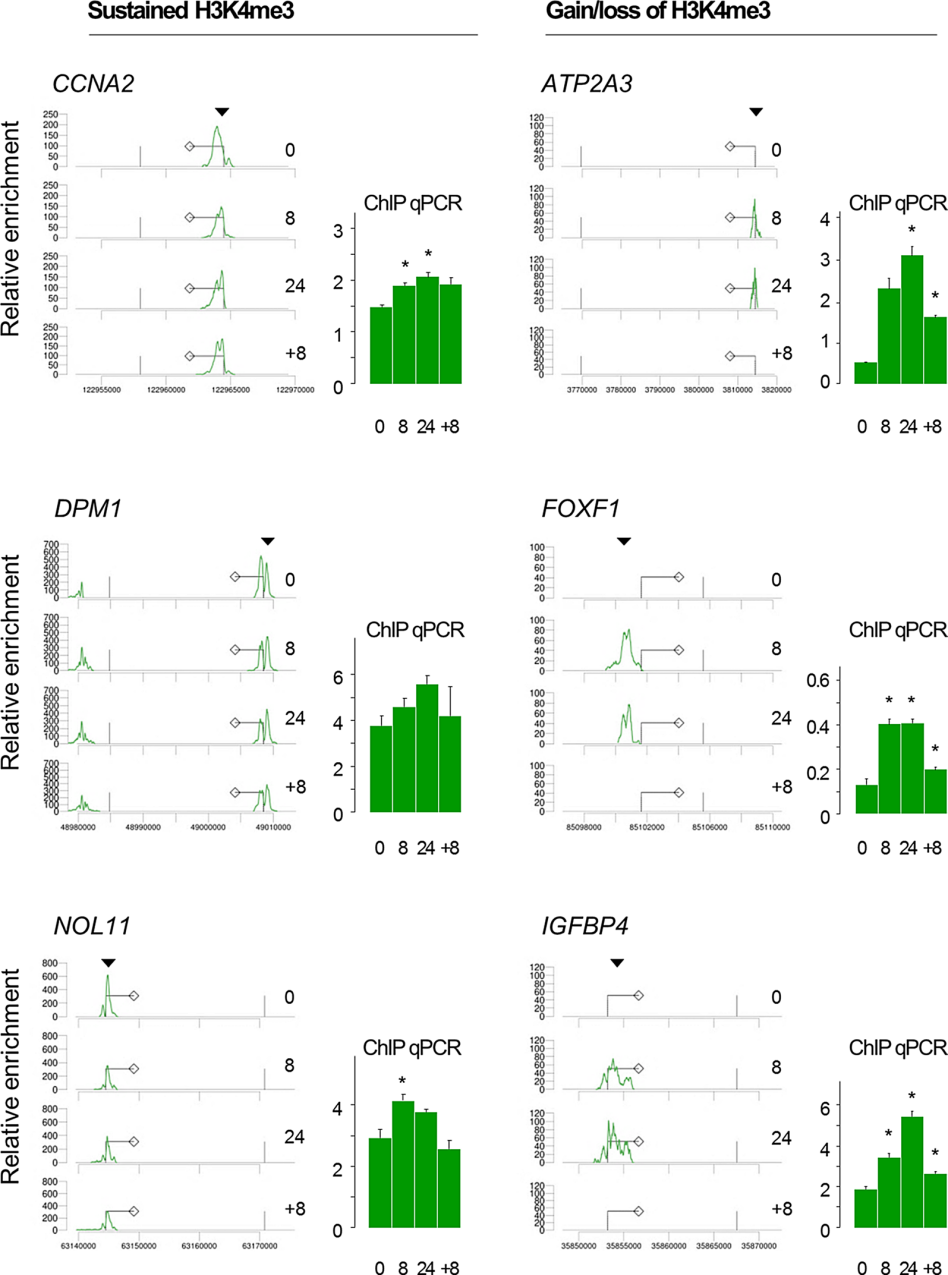
Validation of sustained and dynamic H3K4me3 marking. Bioinformatic calling of enriched regions (gene tracks shown at *left side* for each locus) was validated by ChIP-PCR (*bar plots*; shown at *right side* for each locus) for a number of representative epigenetic profiles. Data for all time points are shown: normoxia (0), hypoxia 8 h (8), hypoxia 24 h (24) and reoxygenation (+8). *CCNA2*, *DPM1* and *NOL11* for genes that show sustained H3K4me3 marking in relation to changes in oxygen pressure (left panels); *ATP2A3*, *FOXF1* and *IGFBP4* for genes that show dynamic modulation of H3K4me3 marking in relation to changes in oxygen pressure (*right panels*); gene-specific ChIP-PCR analyses are part of a biological study published elsewhere [10]. *Diamond symbol* indicates direction of transcription, *black triangle* indicates approximate position of primer set used for validation, and *star symbol* indicates a significant difference compared to *t* = 0 (i.e., normoxia; *p* < 0.05, Wilcoxon signed-rank test)

We and others have previously observed that most H3K4me3 enrichment is located over the transcription start sites (TSSs) of genes. Using a set of 276 epigenetically and transcriptionally invariant genes, the enrichment of H3K4me3 surrounding in TSS (region defined as −1000 bp to +100 bp of the TSS [4]) was summed, yielding sample-specific scaling factors to which each peak in the dataset was scaled. A number of genes with dynamically altered or sustained expression were validated by conventional ChIP-PCR. Oxygen deprivation and/or reoxygenation clearly affected H3K4me3 enrichment at the *ATP2A3*, *FOXF1* and *IGFBP4* loci (Fig. 2), showing a gain in enrichment under hypoxia and a decrease upon reoxygenation, in both the ChIP-seq and the ChIP-PCR results. Although the H3K4me3 enrichment for *CCNA2*, *DPM1* and *NOL11* is relatively sustained in response to hypoxia and subsequent reoxygenation (Fig. 2), the ChIP-PCR results suggest a relatively small change in H3K4me3 enrichment which is not reflected in the ChIP-seq results. A GO enrichment analysis on all 276 genes with sustained H3K4me3 enrichment and high expression suggests that these loci, which encode factors involved in RNA binding, translation and protein transport and localization as main processes, are not subject to transcriptional regulation under fluctuating oxygen levels.

### Hypoxia-induced bivalency is associated with CpG-rich regions near developmental genes

**Fig. 3:**
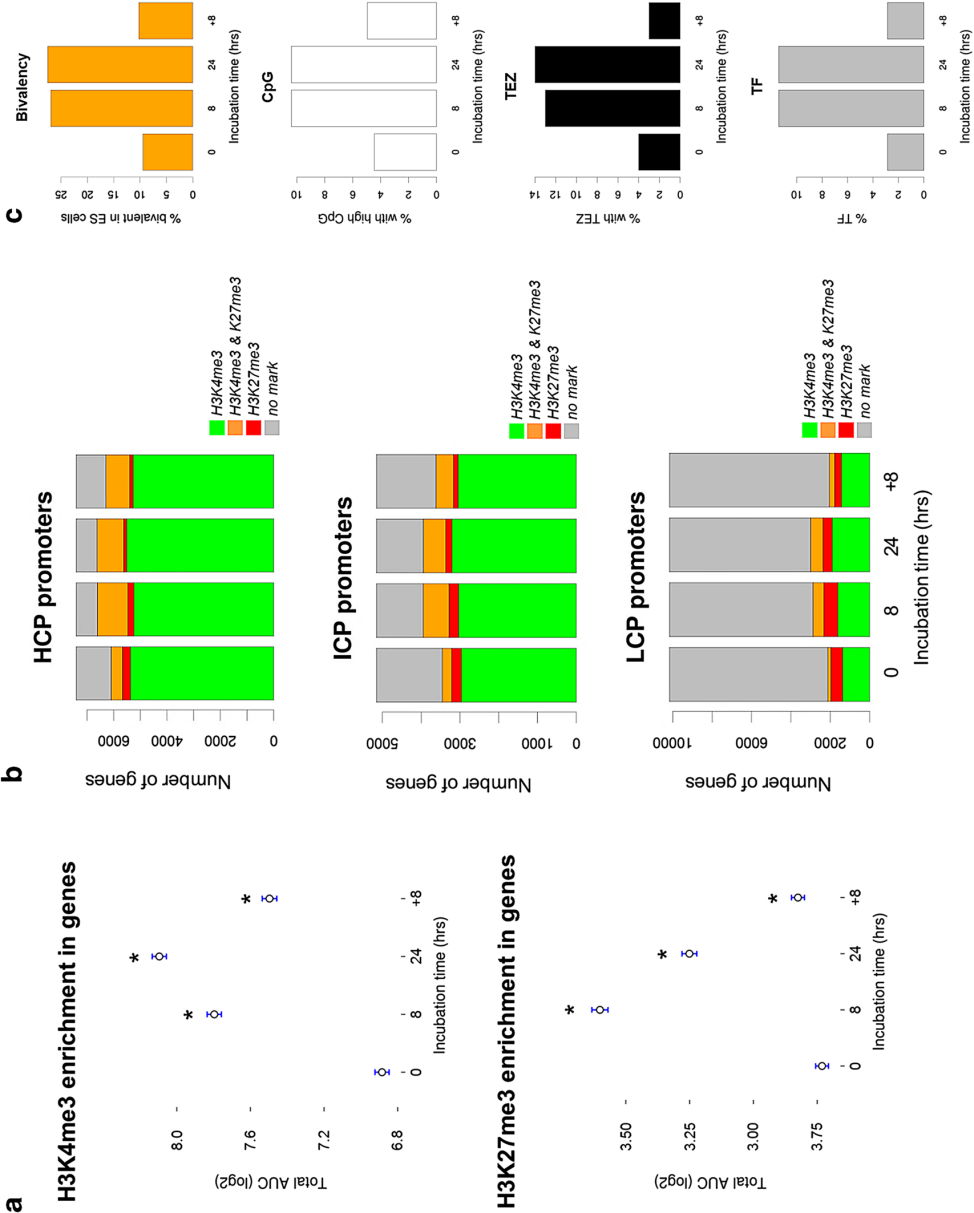
Genomic element enrichment of genes with acquired bivalency under hypoxia. **a** Both H3K4me3 enrichment and H3K27me3 enrichment increase in genes in response to hypoxia, with a decrease upon reoxygenation which is most pronounced for the H3K27me3 mark. The total AUC (log2-transformed) of all ChIP-seq peaks in a gene is represented on the y axis (mean over all genes and standard error are shown). *Star symbol* indicates a significant difference compared to *t* = 0 (i.e., normoxia; *p* < 0.05, Wilcoxon signed-rank test). **b** Distribution of H3K4me3- and H3K27me3-enriched promoters with (*top*) high CpG, (*middle*) intermediate and (*bottom*) low CpG-content. **c** Overlap of bivalent genes in this study with bivalently marked genes in embryonal stem cells (ES cells [27]), transcription factor (TF [25]; in *gray*) genes, genes containing promoter CpG islands (CpG [25]; in *white*) and genes whose TSS coincides with transposon exclusion zones (TEZ [25]; in *black*)

Since the data are normalized and the same threshold is applied across samples, we can reliably assess dynamics of H3K27me3 and H3K4me3 enrichment under hypoxia and subsequent reoxygenation. As illustrated in Fig. 3a, the enrichment of H3K4me3 in genes increases significantly after 8 h (fold change (FC) of 1.88 compared to *t* = 0; *p* < 0.05) and 24 h of hypoxia (FC of 2.32 cf. *t* = 0; *p* < 0.05), with a slight drop upon reoxygenation (FC of 1.51 cf. *t* = 0; *p* < 0.05). Interestingly, H3K27me3 enrichment increases significantly after 8 h of hypoxia (FC of 1.82 cf. *t* = 0; *p* < 0.05), then decreases at 24 h of hypoxia (FC of 1.43 cf. *t* = 0; *p* < 0.05) and then upon reoxygenation drops to comparable levels as observed during normoxia (fold change of 1.08 compared to *t* = 0; *p* < 0.05).

In addition to the dynamic changes observed for the individual trimethyl histone modifications, comparative epigenomic analysis for H3K4me3 and H3K27me3 suggested that in response to oxygen deprivation, the number of bivalently marked genes (*i.e.*, H3K4me3 and H3K27me3) increased (results not shown). Bivalency in embryonic stem cells (ES cells) occurs at key developmental control genes and coincides with genomic cytosine-guanidine dinucleotide (CpG)-content (i.e., CpG islands) [25, 26]. To verify whether this applied to bivalent epigenetic marking in MCF7 cancer cells as well, the prevalence of bivalently marked genes was determined in high CpG-content promoter/enhancer regions (HCPs) versus intermediate and low CpG regions (ICP and LCP, respectively). H3K4me3 preferentially localized to HCP; 5373 HCP, 2963 ICP and 1373 LCP promoters carried H3K4me3 marking at normoxia, corresponding to 73 % of charted HCPs, 58 % of ICPs and 13 % of LCPs, respectively (Fig. 3b). Bivalently marked genes showed a highly similar distribution pattern: 428 HCP, 238 ICP and 155 LCP promoters were positive for both trimethyl histone modifications in control cells (*t* = 0). Relatively few promoters showed H3K27me3 marking, consistent with the relative H3K27me3 enrichment over gene bodies or at the TSS. H3K27me3/promoter distribution was opposite of that of H3K4me3: 296 HCP, 252 ICP and 599 LCP promoters were enriched for H3K27me3 (Fig. 3b). Additionally, we see a high correspondence between bivalent marking in ES cells and CpG content, transcription factor (TF) binding sites and transposon exclusion zones (TEZs); using data from [25, 27] (Fig. 3c).

## Discussion

We have shown that applying a data-driven analysis approach using invariant genes and epigenetically marked regions allows for genome-wide, quantitative data comparison for H3K4me3 and H3K27me3 ChIP-seq data across highly variant experimental conditions. Although opting for data-driven approaches may result in analytical parameter use that may not be applicable to other studies [14], the concepts we apply are. As reported by others [18], one needs to be aware of the biology underlying the data to choose a correct data analysis approach, arguably regardless of the genericity of that choice.

A robust normalization strategy analogous to our approach is spike-in normalization [15–17]. Both methods use a set of common references for data normalization and apply an identical scaling approach based on some cumulative value for these common references [16]. Additionally, others have acknowledged that in the absence of spike-in controls, regions of the genome that have stable quantifiable occupancy through all experimental conditions are appropriate to use as an alternative for ChIP-seq data normalization [13, 16]. Here, due to our dataset lacking spike-in controls, we followed this strategy and, through qPCR validation, demonstrated it to indeed yield robust results. ChIP-seq datasets with and without spike-in controls are both widely generated and available in online repositories, and hence, the choice to apply either approach to a specific dataset would depend solely on the presence or absence of such controls.

Regions with stable enrichment across experimental conditions are primarily located around centromeres. Centromere regions are known for containing a large number of repeats. Repeat region reads will inevitably be mapped to such regions, regardless of their origin, meaning that the high enrichment within these regions might reflect a technical artifact rather than biology. Paired-end sequencing combined with filtering out of reads with ambiguous genomic alignment, as done here, should, however, adequately prevent this from occurring. Also, it has been observed previously that centromeres are heavily enriched for H3K27me3 and other histone modifications [28–31]. ChIP-PCR validation confirmed regions with sustained and dynamic enrichment identified in the ChIP-seq results. Disparities between the PCR and sequencing results, where small changes in enrichment picked up in the former are not reflected in the latter, suggest that mostly large changes in histone trimethylation are picked up in the ChIP-seq analysis. Increasing the sample size could enable a more sensitive analysis of the hypoxia-induced epigenetic changes.

Although the overall number of H3K4me3-, H3K27me3- or H3K4me3/H3K27me3-enriched genes increased in response to hypoxia, their relative association to HCP, ICP and LCP was sustained (Fig. 3b). We and others found a clear increase of bivalent marking at CpG-rich genomic regions [4]. CpG islands are usually hypomethylated (at the DNA level), but instead are marked by trimethylation of histone 3 lysine 4 or lysine 27 [4, 32]; conversely, bivalent domains highly correlate with the presence of CpG islands and may reflect a competition between PRC recruitment (silencing) and transcriptional activation [33]. Key developmental control loci (genes) typically carry numerous TF binding sites, often display high CpG dinucleotide-content and are typically relative transposon free (localize at TEZ). We observed an overlap between the bivalently marked genes in our study and those from a previously published study in ES cells (Fig. 3c; [25, 27]). Thus, congruent with observations in ES cells, hypoxia-induced bivalency in MCF7 cells also occurs at loci encoding factors that control developmental processes, and the association of bivalent marking and CpG-rich regions holds for hypoxia-induced bivalency in MCF7 as well.

## Conclusions

In conclusion, we here demonstrated how a data-driven approach using invariant genes and occupied regions enables genome-wide, quantitative data comparison for H3K4me3 and H3K27me3 ChIP-seq data, integrated with whole-genome transcriptomics in a dynamic biological system. Results are corroborated by published data and current insights regarding epigenetic regulation. *In silico*-identified invariant genomic regions, in addition to dynamic regions, were confirmed through normal ChIP-PCR in vitro, showing that the approach based on dataset-specific invariant regions enables robust analysis of highly dynamic changes in epigenetic marking and gene expression between physiologically distinct states.

## Methods

### Cell culture and experimental conditions

MCF7 (human mammary adenocarcinoma) cells were maintained under standardized culturing conditions (37 °C, 5 % CO2, 100 % humidity). For hypoxic exposure, cells were transferred to a MACS VA500 microaerophilic workstation (Don Whitley Scientific, Shipley, UK) for 8 (acute hypoxia) or 24 h (chronic hypoxia) at <0.02 % O_2_ (5 % H_2_, 5 % CO_2_, residual N_2_). Reoxygenation was achieved by transferring cells to the regular tissue culture conditions (*i.e.*, ambient oxygen levels). Throughout the manuscript, the following definitions are used for each state: normoxia (*t* = 0), hypoxia 8 h (*t* = 8), hypoxia 24 h (*t* = 24) and reoxygenation (*t* =+8).

### Gene expression microarray processing and analysis

RNA for microarray application was isolated using RNeasy mini kit (Qiagen, Hilden, Germany) according to manufacturer’s protocol. Isolations were performed in triplicate. Total RNA samples were analyzed using the Affymetrix expression array platform (Affymetrix Gene Chip 1.0 ST). After scanning, data preprocessing and data analysis were done with R (http://www.R-project.org; version 2.15) using Bioconductor (http://www.bioconductor.org; version 2.11). Data were background-corrected using gcRMA [34]. Two common assumptions for gene expression microarray normalization are that the majority of genes is not differentially expressed between conditions (e.g., from *t* = 0 to *t* = 8) and that there is approximately the same amount of up- and down-regulated genes. Those assumptions did not hold for this study, and hence, a data-driven GRSN rank-invariant normalization [35] was used. Updated ENSEMBL-based probe set annotation was used from Brain Array (http://brainarray.mbni.med.umich.edu/brainarray/). All genes which were not represented on the microarray were not included for further analysis. Functional enrichment of associated genes was performed using the topGO package in R, using the parent–child algorithm [36] with a minimum node-size of 5.

### Chromatin immunoprecipitation (ChIP) assays, qPCR and deep sequencing

Details of the ChIP procedure and ChIP-grade antisera H3K4me3 and H3K27me3 antisera have been described elsewhere [10]; the CBX8 ChIP-grade antiserum was a courtesy of Klaus Hansen (BRIC, Copenhagen, DK) [23]. Input and ChIP samples were sequenced using the 36-bp paired-end protocol on the Illumina Genome Analyser IIx (GAIIx). In order to obtain sufficient sequencing depth, additional lanes were sequenced when necessary. All data obtained from each individual sample were pooled. Paired-end sequencing was chosen as it provides two connected DNA end-tags, enabling reliable identification of enriched regions. In addition, paired-end sequencing is more powerful in identifying enrichment in repeat regions, such as satellite DNA regions near centromeres, and several histone modifications are known to be associated with such regions and as such are of biological interest [28, 37].

### Alignment of ChIP-seq reads and identification of enriched regions

Image processing and base calling were performed using Illumina software tools provided by the manufacturer. Subsequent paired-end genome alignment was performed using Novoalign with Human Genome 18 (HG18) used as a reference genome. Only uniquely aligned reads were used for further analysis. To remove PCR artifacts, all data were collapsed prior to peak calling. To identify enriched regions in the ChIP samples relative to the input control, the peak caller FindPeaks (version 4.0) was used. Although alignment tools in general produce comparable results, FindPeaks [38] is most sensitive in distinguishing enrichment [39] compared to PeakSeq [40], USeq [41] and MACS [42].

Identification of enriched regions requires a null distribution [43], ideally in the form of a sequenced input sample which allows for proper correction of background anomalies, such as amplified regions and variability in shearing of DNA [44]. As we had input samples available, they were used in the enrichment finding process.

For H3K4me3 peak detection, the default settings were used. H3K27me3 enrichment tends to spread and cover an entire locus, a phenomenon termed “blanketing” [4]. This results in data comprising spread-out enrichment signals covering large genomic regions [4, 24, 45] instead of sharp peaks. In order to successfully identify such broad regions of enrichment, while still retaining the advantages of using an input DNA sample as a reference, the *expected sequence distribution* setting in the FindPeaks algorithm was adjusted for H3K27me3, by setting it twice as large as the default settings used for H3K4me. This setting was chosen as we observed that the signal distribution around the TSS for H3K27me3 was approximately twice as broad as the TSS signal of H3K4me3 (Additional file 1: Figure S1).

### H3K27me3 and H3K4me3 ChIP-seq data normalization, ChIP-PCR validation

For H3K27me3 data normalization, an unsupervised approach was applied to identify regions of sustained high enrichment under normoxic and hypoxic conditions. Sustained high enrichment represents regions that have a specific histone marking across the entire population of cells, i.e., maximum enrichment which is unable to increase further. First, WIG files containing peak data were imported into R. All peaks were summarized based on their mean location and maximum height. Next, for each sample (four experimental conditions; i.e., 0, 8 and 24 h of hypoxia and 8 h of reoxygenation) an ACME data object was created [43], and the ACME algorithm was run using a window size of 20 kb and a cutoff of 0.90 corresponding the 90th percentile of all peak heights in a sample. Briefly, based on the cutoff peaks are dichotomized into two classes, positive and negative. Using a sliding window approach, each genomic window is tested for having a significantly higher number of observed positive peaks than expected using a Chi-square test. The window size of 20,000 bp was chosen as it is larger than the observed average peak width, while being small enough to lead to a reasonable specificity. Upon completion of the ACME analysis, only regions with a Chi-square test *p* value smaller than 1 × 10^−5^ in all samples/conditions were considered. The cumulative area under the curve (AUC) for these regions was determined for each sample (four time points). By dividing the four AUC values by the smallest value sample-specific scaling factors were obtained to which all the peaks were scaled.

For H3K4me3 data normalization, we postulated that genes with invariant expression should have invariant H3K4me3 enrichment. Consistent with a previous report [46] that the ratio of H3K4me3 and H3K27me3 enrichment correlates with the degree of gene transcription, we see a similar trend at *t* = 0 (normoxia) (Additional file 1: Figure S2). The robust rank-invariant GRSN normalization application for transcriptomics data allowed us to identify genes with sustained expression across conditions by using the coefficient of variance (CV). Combined with our observation that within our study H3K27me3 modulations appear to have little effect on the differential expression of genes [10], we assumed that H3K4me3 enrichment at the TSS (region defined as −1000 to +100 of TSS [4]) of genes with sustained expression to be invariant. Hence, of all genes with a CV <0.10 and expressed above background level [10] the AUC of all present H3K4me3 peaks in the TSS region (TSS ± 1 kb) was summed. Peaks were subsequently scaled using scaling factors calculated relative to the smallest AUC sum among the samples.

Primers sets used for ChIP-PCR-based validation have been published elsewhere [10]; primer sets used for validation of stable regions were: >*ID:chr3.1|CH:3|1|64757658|64764909*: GAGGCAGGGCTATGCGATAGT (forward) and GTAGGGCGTAAGCAGGGACAC (reverse); >*ID:chr3.2|CH:3|1|64843889|64850613*: TCAATAACTAGGCCACATTGGATC (forward) and ATTGGTACAAGACCTCCTGGCTT (reverse).

### Linking of microarray gene expression data and H3K27me3 and H3K4me3 ChIP-seq data at gene level

 The microarray gene expression data and the H3K27me3 and H3K4me3 enrichment ChIP-seq data were integrated using the latest ENSEMBL genome annotation. For the identification of H3K27me3- and/or H3K4me3-enriched genes, we defined a gene as the region between its 5′ (most upstream TSS) and 3′ (last exon) end plus 5 kb regulatory regions up and downstream, respectively. A gene was called enriched or marked when there was at least one ChIP-seq peak above background level present within this region as determined by the enrichment finding procedure. Gene-based enrichment levels are the total AUC of all peaks present in the gene region.

### Statistical analyses

All differences in H3K4me3 and H3K27me3 enrichment levels, for both the standard ChIP-PCR results and ChIP-sequencing results, were tested statistically by the Wilcoxon signed-rank test. *p* values for Spearman correlations were computed via the asymptotic *t* approximation. In all cases, a nominal *p* value smaller than 0.05 was considered statistically significant.

## Additional file

**Additional file 1: Figure S1.** (*Top*) H3K4me3 peak intensity density distribution proximal to the TSS in relation to oxygen deprivation and reoxygenation. (*Bottom*) H3K27me3-distribution proximal to the TSS in relation to oxygen deprivation and reoxygenation. Legend: t=0: normoxia; t=8: 8 hours of hypoxia; t=24: 24 hours of hypoxia; t=+8: 8 hours of subsequent reoxygenation. These figures and underlying data have also been published in an accompanying paper [10]. **Figure S2.** Relation between the ratio of H3K4me3 and H3K27me3 enrichment at the transcription start site for each gene with its associated expression level at 0 hours of hypoxia (i.e. t=0, normoxia). Higher enrichment is associated with higher expression, as observed previously [46].

## Abbreviations

ChIP-PCR: Chromatin immunoprecipitation-polymerase chain reaction
ChIP-seq: Chromatin immunoprecipitation-deep sequencing
CBX8: Chromobox protein 8
CpG: Cytosine guanidine dinucleotide
ES cells: Embryonic stem cells
H3K27me3: Histone 3 lysine 27 trimethylated
H3K4me3: Histone 3 lysine 4 trimethylated
HCP: High CpG-content promoter/enhancer region
ICP: Intermediate CpG-content promoter/enhancer region
LCP: Low CpG-content promoter/enhancer region
PRC1: Polycomb repressive complex 1
TF: Transcription factor
TEZ: Transposon exclusion zone
TSS: Transcription start site
