# Supplementary material for: Quantitative analysis of ChIP-seq data uncovers dynamic and sustained H3K4me3 and H3K27me3 modulation in cancer cells under hypoxia
Source: Epigenetics Chromatin. 2016 Nov 1;9:48. doi: 10.1186/s13072-016-0090-4 (PMC5090954; doi:10.1186/s13072-016-0090-4)
Supplement: Supplementary file 1 — Additional file 1: Figure S1. (Top) H3K4me3 peak intensity density distribution proximal to the TSS in relation to oxygen deprivation and reoxygenation. (Bottom) H3K27me3-distribution proximal to the TSS in relation to oxygen deprivation and reoxygenation. Legend: t=0: normoxia; t=8: 8 hours of hypoxia; t=24: 24 hours of hypoxia; t=+8: 8 hours of subsequent reoxygenation. These figures and underlying data have also been published in an accompanying paper [10]. Figure S2. Relation between the ratio of H3K4me3 and H3K27me3 enrichment at the transcription start site for each gene with its associated expression level at 0 hours of hypoxia (i.e. t=0, normoxia). Higher enrichment is associated with higher expression, as observed previously [46]. [file 13072_2016_90_MOESM1_ESM.docx]

**
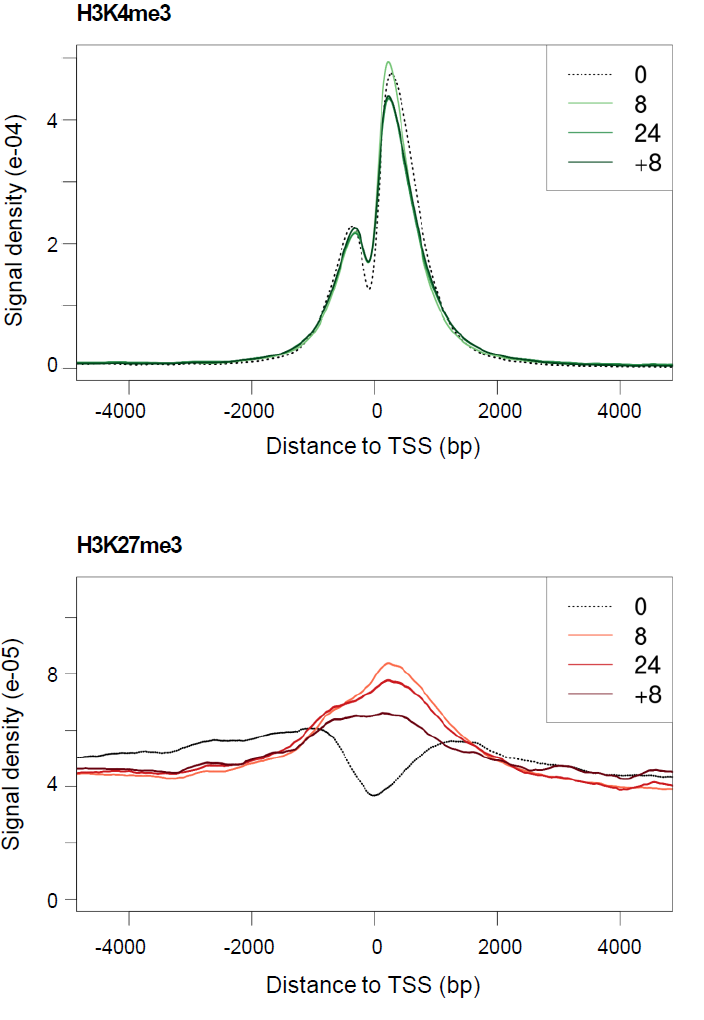
**

**Figure S1.** (*Top*) H3K4me3 peak intensity density distribution proximal to the TSS in relation to oxygen deprivation and reoxygenation. (*Bottom*) H3K27me3-distribution proximal to the TSS in relation to oxygen deprivation and reoxygenation. Legend: t=0: normoxia; t=8: 8 hours of hypoxia; t=24: 24 hours of hypoxia; t=+8: 8 hours of subsequent reoxygenation. These figures and underlying data have also been published in an accompanying paper (Prickaerts *et al.*, *Epigenetics & Chromatin, 2016*).

**
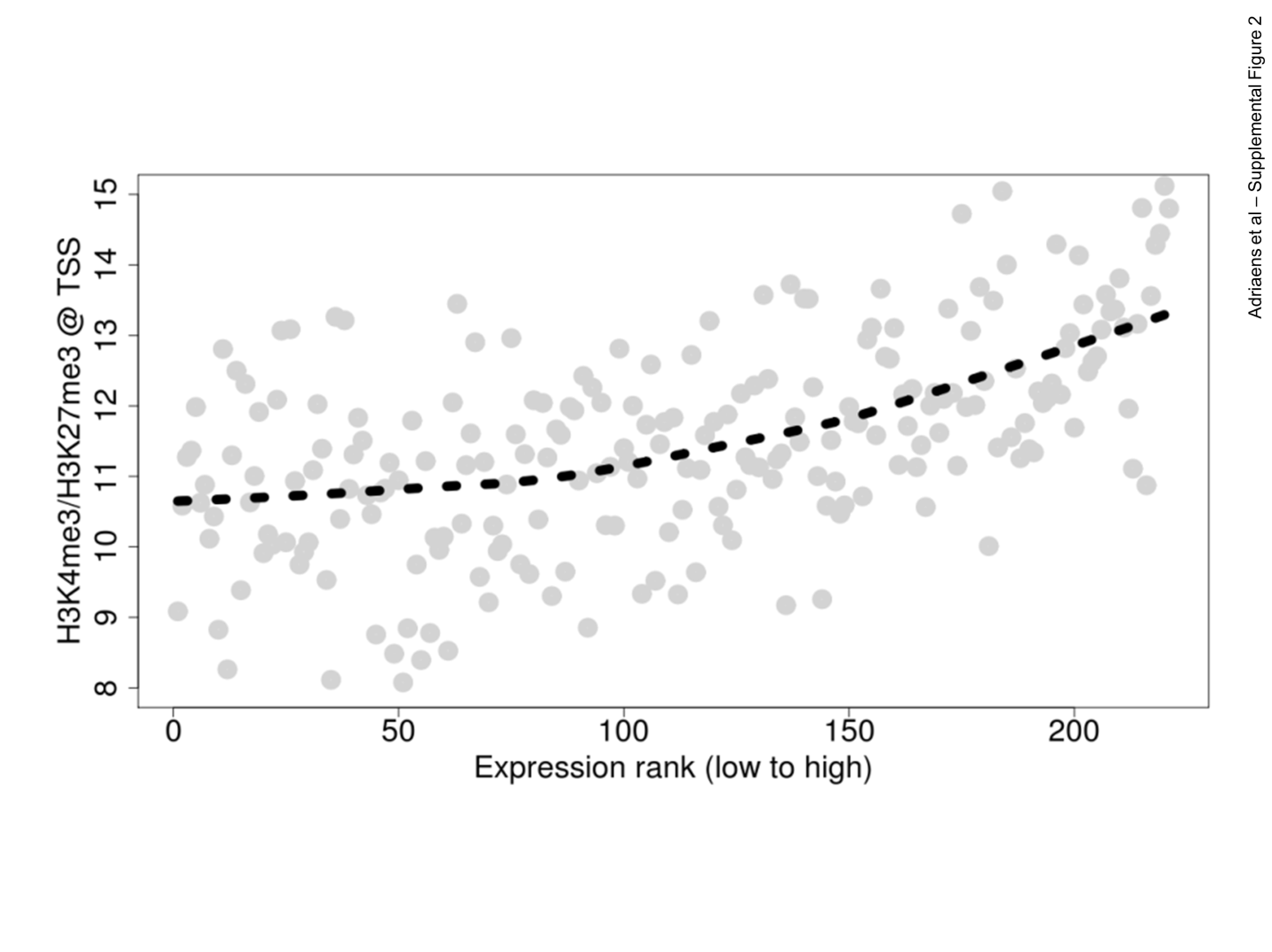
**

**Figure S2.** Relation between the ratio of H3K4me3 and H3K27me3 enrichment at the transcription start site for each gene with its associated expression level at 0 hours of hypoxia (i.e. t=0, normoxia). Higher enrichment is associated with higher expression, as observed previously ([De Gobbi *et al.* 2011](#_ENREF_11)).
